# Association Between the rs4680 Polymorphism of the COMT Gene and Personality Traits among Combat Sports Athletes

**DOI:** 10.5114/jhk/168789

**Published:** 2023-07-06

**Authors:** Kinga Humińska-Lisowska, Krzysztof Chmielowiec, Jolanta Chmielowiec, Aleksandra Strońska-Pluta, Aleksandra Bojarczuk, Magdalena Dzitkowska-Zabielska, Beata Łubkowska, Michał Spieszny, Olga Surała, Anna Grzywacz

**Affiliations:** 1 Faculty of Physical Education, Gdansk University of Physical Education and Sport, Gdansk, Poland.; 2 Institute of Sports Sciences, University of Physical Education in Krakow, Krakow, Poland.; 3 Department of Hygiene and Epidemiology, Collegium Medicum, University of Zielona Góra, Zielona Góra, Poland.; 4 Independent Laboratory of Health Promotion, Pomeranian Medical University in Szczecin, Szczecin, Poland.; 5 Institute of Sport, National Research Institute, Warszawa, Poland.

**Keywords:** catecholamine neurotransmitters, mentality, genetic predispositions, sport

## Abstract

Physical performance has been the focus of studies examining genetic influences in martial arts. There has been little quantitative analysis of the interaction between psychological traits and gene variants in athletes. This study aimed to determine whether the rs4680 polymorphism of the COMT gene (catechol-O-methyltransferase) was linked to other sports phenotypes such as temperament, mental toughness, and stress tolerance. In our study, we concentrated on the case-control analysis of athletes in the aspect of their personality traits in association with the COMT gene polymorphism. Participants comprised 258 combat sports athletes and 278 healthy male individuals as a control group. Psychometric properties were assessed with the Revised Temperament and Character Inventory (TCI-R). COMT polymorphism testing was performed using real-time PCR. We found a statistically significant effect of a complex factor COMT rs4680 genotype with combat athletes/controls and novelty seeking (F_2,530_ = 5.958, p = 0.0028, η^2^ = 0.022), self-management (F_2,530_ = 6.772, p = 0.0012, η^2^ = 0.025), and with self-transcendence skills (F_2,530_ = 9.387, p = 0.00009, η^2^ = 0.034). The results are important for encouraging further studies on the genetic makeup of athletes in conjunction with personality traits. Due to the multigene and multifactorial nature of determinants of sports predispositions, we propose to take into account also other features, especially when studying genes related to cerebral neurotransmission. It is a holistic departure, and it clearly illustrates the relationship between the given characteristics of an athlete.

## Introduction

Some athletes exhibit exceptional achievements in certain sports, with equal and/or similar training and physical conditions. Importantly, among the most crucial elements of success is an athlete's natural talent. However, every person may achieve a certain degree of physical fitness differently due to genetic diversity. Furthermore, a large number of a specific abilities and characteristics are genetically predetermined. Motor coordination processes occur mainly through neurophysiological mechanisms of control and regulation of sensorimotor, perceptual, intellectual, proprioceptive, and kinesiological functions. They rely on the central nervous system, the sensory organs (receptors), and the muscular system (effectors), all of which are influenced by genetic and environmental factors (Kochanowicz et al., 2015). Developments in DNA sequencing technology have led to a better understanding of the role of genetic variants in athletic performance, resulting in the development of “sports genomics”. Over the past 20 years, intensive research has identified as many as 185 genetic markers that can be linked to an athlete's elite status (Maciejewska-Skrendo et al., 2019; Youn et al., 2021).

One of the best known genes involved in the metabolism of catecholamine neurotransmitters (e.g., adrenaline, noradrenaline, or dopamine) is the *COMT* gene (catechol-O-methyltransferase) (Bastos et al., 2017; Craddock et al., 2006; Perkovic et al., 2018). *COMT* is located at “22q11.1 – q11.2” and has a size of approximately 27 Kbp, with 345 polymorphisms identified (Chen et al., 2021; Harrison and Tunbridge, 2008). The magnesium enzyme (Mg2^+^) transfers the COMT methyl group from S-adenosyl-L-methionine (SAM) to one of the catechol hydroxyl groups. The O-methylation is important in the inactivation of catecholamine neurotransmitters and catechol hormones such as dopamine (DA) and norepinephrine (NA) (Bastos et al., 2017). In humans, there are two isoforms of COMT, expressed from different promoters: the membrane-bound form (MB-COMT) and the soluble form (S-COMT). The first isoform controls extracellular DA levels in the prefrontal cortex and is mostly expressed in brain neurons. Its function was revealed in the late 1950s, yet researchers have been also interested in the role of the COMT enzyme in additional pathways and illnesses. COMT has been studied from the perspective of neuropsychiatric conditions and the *neural underpinnings of cognitive functions*, emotion, behavior, sleep and pain mechanisms, as well as addictive behavior and neurodegeneration (Bastos et al., 2017; Machoy-Mokrzyńska et al., 2019). It has been stated that athletic talent may be significantly influenced by the COMT enzyme activity (Zmijewski et al., 2021).

The rs4680 *COMT* single nucleotide polymorphism (SNP) is one of the most frequently analyzed variants of exon 4, as rs4680 takes an active part in the prefrontal cortex's enzymatic activity and cognitive function (Haraldsson et al., 2010). SNP rs4680, due to a functional change from G to A nucleotide, can cause a valine (Val) to methionine (Met) substitution at codon 158 (in membrane-bound COMT) or codon 108 (insoluble COMT), thereby generating changes in the enzyme's capacity (Craddock et al., 2006; Harrison and Tunbridge, 2008). The Val variant has almost two- to four-times higher enzymatic activity than the Met variant (Chen et al., 2021; Haraldsson et al., 2010). A polymorphism that causes the amino acid encoded by codon 158 of the *COMT* gene, substituted from valine to methionine (*COMT* Val158Met), has been shown to reduce the enzyme's activity by one-third to one-quarter of that of valine-type *COMT* (Savitz et al., 2006), therefore enhancing extracellular DA levels. It has been proposed that the *COMT* Val158Met SNP may be related to individual differences in emotional reactions, motivation, and executive control ability (Jaspar et al., 2014).

It is generally agreed that athletes’ personality traits, including emotional resilience, persistence and stress management, are qualities enabling them to succeed in sports (Youn et al., 2021). However, research on psychogenetic factors affecting elite martial arts athletes' mental or emotional strength has been scarce. Nevertheless, previous studies on genetic factors of emotional and psychological traits suggest that genetic variants may influence various phenotypic traits associated with elite athletic performance (Youn et al., 2021; Humińska-Lisowska et al., 2022).

Most research in martial arts has emphasized that genetics contribute to athletic or physical performance (Cieszczyk et al., 2011; Guilherme et al., 2019, 2021; Ribas et al., 2017; Ruzic et al., 2023). Future investigations should search for DNA sequences related to other sporting phenotypes, i.e., the ability to cope with stress, mental strength, and attitude. Our study focused on the case-control analysis of athletes in terms of personality traits associated with the COMT gene polymorphism.

## Methods

### Participants

A total of 536 Caucasian men from the same area of Poland were examined. The study group consisted of 258 combat sports athletes (mixed martial arts (MMA), n = 85; judo, n = 53; boxing, n = 51; karate, n = 21; kickboxing, n = 34; wrestling, n = 14) with no previous history of addiction or psychosis. The control group included 278 healthy volunteers who were not competitive athletes (Table 1). The study group was considered “sub-elite” (participants of international or national sports competitions with a minimum of five years of training). Various methods were used to obtain the study sample, including targeting national teams and providing information to national coaching personnel and athletes attending training camps. All study participants were of the Caucasian origin to avoid differences in allele frequencies due to systematic differences in ancestry arising from population stratification.

**Table 1:** Anthropometric characteristics of the study participants.

	Combat sports athletes n = 258	Controls n = 278
Age	26.01 ± 8.30	22.93 ± 4.81
Body mass	79.67 ± 13.68	81.28 ± 10.69
Body height	178.46 ± 7.31	181.73 ± 6.07
BMI	24.57 ± 4.18	24.28 ± 3.93

### Psychometric Tests

Psychometric tests such as the Revised Temperament and Character Inventory (TCI-R) were conducted. Temperament refers to individual differences in perceptual and skill-based habits that are regulated by the limbic system and measured by independently inherited dimensions which are moderately stable throughout life. The TCI-R is a self-administered survey that assesses the four dimensions of temperament (novelty seeking (NS), the tendency to react and depend on reinforcements (reward dependence—RD), pain avoidance (harm avoidance—HA), and perseverance (PS)) and the three components of the higher-order character (cooperation, self-direction, and transcendence) as previously described (Harrison and Tunbridge, 2008; Zmijewski et al., 2021). The licensed TCI-R test, Polish version from 2018, was performed and interpreted by a trained psychologist (Niewczas et al., 2021).

### Statistical Analysis

The distribution of *COMT* rs4680 genotype frequencies was tested for the Hardy-Weinberg equilibrium (HWE) using HWE software (https://wpcalc.com/en/equilibrium-hardy-weinberg/; access date: 15 June 2021). The examined variables were not normally distributed. The Mann-Whitney U-tests were used to evaluate variations in the studied characteristics: novelty seeking, reward dependence, cooperative ability, self-transcendence ability, harm avoidance, and self-management. Not all conditions needed for ANOVA were met. For some dependent variables, the normal distribution assumption was not met. Nevertheless, the variance remained constant (Levene's test, *p* > 0.05). Because of the large sample size, a multivariate 2 × 3 factor ANOVA was used. The relationship between novelty seeking, harm avoidance, reward addiction, self-management, cooperation abilities, and self-transcendence skills results and combat athletes and the control group, and the *COMT* rs4680 polymorphism (personality traits x control and combat athletes subjects x genetic feature) was examined. For statistically significant ANOVA results, the post hoc least significant difference (LSD) test was used.

The chi-square and Cochran-Armitage trend tests were applied to determine associations of *COMT* rs4680 with combat athletes. Statistical analyses were conducted using STATISTICA 13 (Tibco Software Inc., Palo Alto, CA, USA) for Windows (Microsoft Corporation, Redmond, WA, USA) and the CATT package in R to conduct the Cochran-Armitage trend test.

## Results

Genotype association analysis of *COMT* rs4680 polymorphisms in combat sports athletes and controls indicated statistically significant differences in the co-dominant model in frequencies of genotypes for *COMT* rs4680 (G/G 24.42% vs. G/G 21.58%; G/A 53.49% vs. G/A 44.96%; A/A 22.09% vs. A/A 33.45%; χ^2^ = 8.621; *p* = 0.0134, Table 2). Combat sports athletes and controls showed statistically significant differences in the frequency of alleles for the *COMT* rs4680 (G 51.16% vs. G 44.06%, A 48.84% vs. A 55.94%, χ^2^ = 5.410, *p* = 0.0201, Table 2).

**Table 2:** The frequency of genotypes and alleles of *COMT* rs4680 in combat sports athletes and controls.

	Combat sports athletes	Controls	Co-dominant model χ^2^ (*p* value)	OR (95% Confidence, *p* value )	Additive model Cochran-Armitage trend test Z (*p* value)
*COMT* rs4680					
	*n* = 258	*n* = 278
G/G	63 (24.42%)	60 (21.58%)	8.621 (0.0134)*		−2.307 (0.0211)
G/A	138 (53.49%)	125 (44.96%)		1.05 (0.68–1.61, *p* = 0.4093)	
A/A	57 (22.09%)	93 (33.45%)		0.58 (0.36–0.94, *p* = 0.0145)*	
G	264 (51.16%)	245 (44.06%)	5.410 (0.0201)		
A	252 (48.84%)	311 (55.94%)			

p: statistical significance, χ^2^: Chi^2^ test result, n: number of subjects, * significant statistical difference, OR: Odds Ratio, G/G and A/A: genotypes (homozygotes), G/A: genotype (heterozygote), G and A: alleles

**Table 3:** Analysis of novelty seeking, harm avoidance, reward dependence, self-management, ability to cooperate, and self-transcendence results in combat sports athletes and controls.

	Combat sports athletes	Controls	U Mann-Whitney Z	*p* value
	(n = 258) M ± SD	(n = 278) M ± SD		
Novelty Seeking	20.20 ± 4.79	19.92 ± 4.34	0.667	0.5045
Harm Avoidance	9.64 ± 4.83	11.46 ± 4.63	−4.513	0.0000*
Reward Dependence	10.03 ± 3.03	10.47 ± 2.87	−1.449	0.1471
Self-Management	26.10 ± 4.40	23.73 ± 4.81	5.715	0.0000*
Cooperative Abilities	20.59 ± 4.54	19.92 ± 4.60	1.834	0.0667
Self-Transcendence Skills	7.12 ± 3.47	6.92 ± 3.39	0.484	0.6286

M: mean, SD: standard deviation, U Mann-Whitney Z-test. * statistically significant between-group difference

Table 3 displays mean and standard deviation data for cooperation abilities, self-transcendence skills, novelty seeking, harm avoidance, reward dependence, and self-management skills in a group of combat athletes and controls. Compared to the control group, athletes had considerably higher scores in self-management (M 26.10 vs. M 23.73, Z = 5.715, *p* < 0.0001) and lower results considering harm avoidance (M 9.64 vs. M 11.46, Z = −4.513, *p* = 0.019, Table 3).

### Novelty Seeking

**Figure 1:**
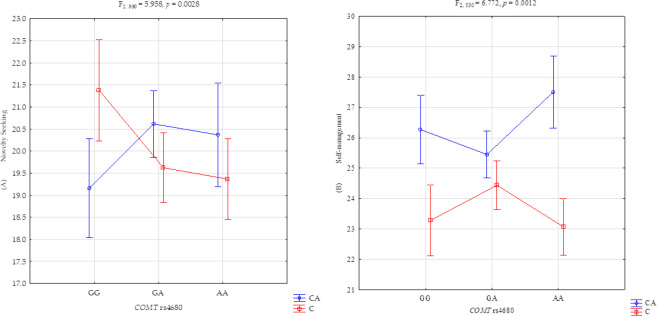
Interaction between combat sports athletes (CA)/controls (C), *COMT* rs4680 and (A) the novelty seeking scale, and (B) self-management scale. GG and AA: genotypes (homozygotes), GA: genotype (heterozygote)

The 2 × 3 factorial ANOVA showed a statistically significant effect of the complex factor *COMT* rs4680 genotype with combat sports athletes/controls and with novelty seeking (F_2,530_ = 5.958, *p* = 0.0028, η^2^ = 0.022) (Table 4, Figure 1). Power calculation assessment revealed that the sample size in the present study had more than 87% power to detect the complex factor of combat athletes/controls x *COMT* rs4680 and their interaction effect (about 2% of the phenotype variance).

**Table 4:** The results of 2 × 3 factorial ANOVA (interaction) for combat sports athletes and controls, incorporating novelty seeking, harm avoidance, reward dependence, self-management, ability to cooperate, self-transcendence skills results and *COMT* rs4680.

STAI /NEO Five Factor Inventory	Group	rs4680			ANOVA (interaction)		
		G/G (n = 123) M ± SD	G/A (n = 263) M ± SD	A/A (n = 150) M ± SD	Combat athletes /controls x *COMT* rs4680 F (*p* value)	ɳ^2^	Power (alfa = 0.05)
Novelty Seeking	Combat sports athletes; n = 258	19.15 ± 4.20	20.62 ± 4.87	20.37 ± 5.13	F_2,530_ = 5.958 ( *p* = 0.0028)*	0.022	0.8789
	Controls; n = 278	21.38 ± 4.25	19.62 ± 4.39	19.37 ± 4.17			
Harm Avoidance	Combat sports athletes; n = 258	9.69 ± 5.01	9.65 ± 4.84	9.56 ± 4.70	F_2,530_ = 0.065 (*p* = 0.9373)	0.0002	0.059
	Controls; n = 278	11.43 ± 4.77	11.36 ± 4.53	11.61 ± 4.71			
Reward Dependence	Combat sports athletes; n = 258	9.92 ± 2.77	10.02±3.20	10.16 ± 2.85	F_2,530_ = 0.450 (*p* = 0.6377)	0.002	0.123
	Controls; n = 278	10.72 ± 2.84	10.22 ± 3.16	10.64 ± 2.46			
Self-Management	Combat sports athletes; n= 258	26.27 ± 4.21	25.45 ± 4.75	27.51 ± 3.33	F_2,530_ = 6.772 (*p* = 0.0012)*	0.025	0.918
	Controls; n = 278	23.28 ± 4.37	24.44 ± 4.84	23.07 ± 4.95			
Cooperative Abilities	Combat sports athletes; n = 258	20.11 ± 4.52	20.62 ± 4.71	21.07 ± 4.14	F_2,530_ = 0.743 (*p* = 0.4762)	0.003	0.176
	Controls; n = 278	20.27 ± 4.54	19.73 ± 4.40	19.94 ± 4.92			
Self-Transcendence Skills	Combat sports athletes; n = 258	6.65 ± 3.34	6.98 ± 3.31	7.98 ± 3.88	F_2,530_ = 9.387* (*p* = 0.00009)	0.034	0.979
	Controls; n = 278	8.60 ± 4.06	6.50 ± 3.08	6.42 ± 2.96			

M: mean, SD: standard deviation, * statistically significant differences, G/G and A/A: genotypes (homozygotes), G/A: genotype (heterozygote)

Post hoc LSD analysis showed a significantly lower novelty seeking score in combat sports athletes for the *COMT* rs4680 polymorphic variant (G/G M = 19.159) compared to controls (G/G M = 21.383 *p* = 0.0067) (Table 5, Figure 1).

**Table 5:** Post hoc LSD test of interactions between combat sports athletes/controls, *COMT* rs4680 and the novelty seeking scale, self-management and self-transcendence skills.

**Novelty Seeking scale**						
***COMT* rs4680**	{1} 19.159	{2} 21.383	{3} 20.616	{4} 19.624	{5} 20.368	{6} 19.366
GG Combat sports athletes {1}		0.0067*	0.0348*	0.5063	0.1445	0.7796
GG Controls{2}			0.2736	0.0137*	0.226137	0.0073*
GA Combat sports athletes {3}				0.0766	0.7286	0.0401*
GA Controls {4}					0.3041	0.6770
AA Combat sports athletes {5}						0.1886
AA Controls {6}						
**Self-Management**						
*COMT* rs4680	{1} 26.270	{2} 23.283	{3} 25.449	{4} 24.440	{5} 27.509	{6} 23.075
GG Combat sports athletes {1}			0.0003*	0.2388	0.0099*	0.1391	0.00002*	
GG Controls{2}				0.0023*	0.1081	0.000001*	0.7837	
GA Combat sports athletes {3}					0.0746	0.0044*	0.0001*	
GA Controls{4}						0.00003*	0.0298*	
AA Combat sports athletes {5}							0.000000*	
AA Controls {6}								
**Self-Transcendence Skills**						
*COMT* rs4680	{1} 6.6508	{2} 8.6000	{3} 6.9783	{4} 6.5040	{5} 7.9825	{6} 6.4194
GG Combat sports athletes {1}		0.0014*	0.5224	0.7778	0.0308*	0.6735
GG Controls{2}			0.0019*	0.0001*	0.3215	0.0001*
GA Combat sports athletes {3}				0.2542	0.0586	0.2162
GA Controls {4}					0.0062*	0.8543
AA Combat sports athletes {5}						0.0059*
AA Controls {6}						

* statistically significant differences, M: mean, GG and AA: genotypes (homozygotes), GA: genotype (heterozygote)

### Self-Management

The results of 2 × 3 factorial ANOVA of combat sports athletes and control subjects were significant for self-management (F_1,530_ = 44.61, *p* < 0.0001, η^2^ = 0.078). Our sample had 99% power to detect the effects of the studied self-management and the interaction effect (approximately 8% of the phenotype variance). Additionally, we observed a statistically significant effect of the complex factor *COMT* rs4680 genotype with combat sports athletes/controls and with self-management (F_2,530_ = 6.772, *p* = 0.0012, η^2^ = 0.025) (Table 4, Figure 1). Our sample had more than 92% power to discern the complex factor of combat sports athletes/controls x *COMT* rs4680 and the interaction effect (around 2% of the phenotype variance).

Post hoc LSD analysis revealed that the *COMT* rs4680 polymorphic variant G/G M = 26.270 had a significantly lower self-management score in combat sports athletes than in controls G/G M = 23.283 (*p* = 0.0003) and A/A M = 23.075 (*p* = 0.0001). The same was true for self-management in combat sports athletes regarding variant G/A M = 25.449 compared to controls for A/A M = 23.075 (*p* = 0.0001) variants. Additionally, lower scores of self-management in combat sports athletes regarding A/A variant M = 27.509 versus controls A/A M = 23.075 (*p* < 0.0001) were detected (Table 5, Figure 1).

### Self-Transcendence

The results of 2 × 3 factorial ANOVA of the *COMT* rs4680 genotype were found significant for self-transcendence skills (F_2,530_ = 3.035, *p* = 0.0489, η^2^ = 0.011). Power calculation showed that our sample had 59% power to detect the influence of the studied self-transcendence skills and their interaction effect (about 1% of the phenotype variance) in the *COMT* rs4680 genotype. Moreover, we noticed a statistically significant relationship of the complex factor *COMT* rs4680 genotype with combat athletes/control and self-transcendence skills (F_2,530_ = 9.387, *p* = 0.00009, η^2^ =0.034) (Table 4). Our sample had more than 98% power to detect the complex factor of combat sports athletes/controls x *COMT* rs4680 and their interaction effect (about 3.4% of the phenotype variance).

Post hoc LSD analysis showed a significantly lower score of self-transcendence skills in combat sports athletes for the *COMT* rs4680 polymorphic variant G/G M = 6.650 compared to controls for G/G M = 8.600 variants (*p* = 0.0014). The same was true for self-transcendence skills in combat sports athletes regarding variant G/A M = 6.9783 compared to controls for G/G M = 8.6000 variants (*p* = 0.0019). In addition, A/A variant M = 7.982 had lower self-transcendence scores in combat sports athletes than in controls (A/A M = 6.419, *p =* 0.0001, and G/G M = 8.600, *p* = 0.0001; Table 5).

## Discussion

Our study focused on a case-control analysis of martial arts athletes with personality traits associated with the *COMT* gene rs4680 polymorphism. The current study found a significant association between the *COMT* rs4680 polymorphism and the psychological profile in combat athletes. We discovered a significant effect of a complex factor *COMT* rs4680 genotype on combat sports athletes/controls and novelty seeking, self-management, and self-transcendence skills. The *COMT* gene is responsible for the degradation of catecholamines. This has been linked to genotypes associated with dopamine availability in the brain. *COMT* gene variants are engaged in a number of psychological roles, e.g. cognition, anxiety, and stress response (Nogueira et al., 2019). However, *COMT* gene results are controversial. According to Leźnicka et al. (2017, 2018), no significant relationship (*p* = 0.286 and *p* = 0.43) between the *COMT* rs4680 GG genotype and elite martial arts athletes exists. This implies that the perception of pain is the same in both the martial arts athletes and non-athletes. In contrast, Tatar et al. (2020) demonstrated a significantly higher frequency of the GG (fighter) phenotype in combat sports athletes compared to the control group (*p* = 0.003). This suggests that in combat sports, a fighter's genotype may be important. In our research, we observed a statistically significant effect of a complex factor of rs4680 of the *COMT* genotype on combat sports athletes/controls regarding novelty seeking (F_2,530_ = 5.958, *p* = 0.0028, η^2^ = 0.022), self-management (F_2,530_ = 6.772, *p* = 0.0012, η^2^ = 0.025), and self-transcendence skills (F_2,530_ = 9.387, *p* = 0.00009, η^2^ = 0.034). Our genotype association analysis of *COMT* rs4680 polymorphisms in combat sports athletes and controls indicated statistically significant differences in the co-dominant model in frequencies of genotypes for *COMT* rs4680 (G/G 24.42% vs. G/G 21.58%; G/A 53.49% vs. G/A 44.96%; A/A 22.09% vs. A/A 33.45%; χ^2^ = 8.621; *p* = 0.0134). Combat sports athletes and controls showed statistically significant differences in the frequency of alleles for the *COMT* rs4680 (G 51.16% vs. G 44.06%, A 48.84% vs. A 55.94%, χ^2^ = 5.410, *p* = 0.0201).

While inherited genetic elements can significantly affect an athlete's character or potential, athletic success may also be affected by factors arising from the environment. It would be preferable to use genetic information to build tailored training methods that boost athletes' talent or attributes and prevent potential injuries rather than focus on the abilities of only elite athletes because many factors affect athletic performance (Youn et al., 2021). Numerous attempts to determine the potential implications of the *COMT* rs4680 polymorphism for neuropsychiatric disorders have been made because of the polymorphism's relatively high frequency and critical function in regulating catechol-amine catabolism (Bilder et al., 2004; Srivastava et al., 2021). The rs4680 polymorphism of the *COMT* gene leads to a substitution of Val with Met at codon 158. This in turn leads to the fact that the enzyme is prone to conformational changes at the active site and clustering of the polypeptide at physiological temperature. This results in decreased enzymatic activity in Met allele carriers, while higher activity is observed in Val allele carriers (Bilder et al., 2004). Met/Met homozygotes are responsible for a 3–4-fold decrease in COMT activity in Val/Val carriers (Weinshilboum et al., 1999), and higher baseline fat levels, compared to Val/Val carriers, with heterozygotes showing an average activity (Switala et al., 2022). The frequency of the Met allele varies by ethnicity, ranging from 0.01 to 0.62, e.g., 0.49–0.54 in Caucasians, 0.49 in Southwest Asia, 0.18–0.3 in East Asians and 0.03–0.04 in African Americans and Africans (Bastos et al., 2017). The Met genotype, according to Bilder et al. (2004), is associated with higher tonic dopamine levels, reciprocal dopamine phase reductions in the subcortical areas, and increased D1 receptor transmission in the cortex. As a result, the activation levels of brain networks that underpin critical components of working memory and executive processes may be more stable, but less flexible. Depending on the phenotype and many endogenous and environmental factors, these impacts can be positive or negative (Bilder et al., 2004). Some authors have speculated that chronic physical exertion, as observed in ultra-endurance athletes, manifests in Met/Met homozygotes exhibiting greater novelty seeking traits, corroborating the hypothesis of a relationship between personality traits and *COMT* Val158Met heterozygotes (rs4680) (van Breda et al., 2015; Zmijewski et al., 2021). In a study conducted by Leźnicka et al. (2018), the relationship between genetic diversity and temperamental features was also established. Notably, associations between the functional polymorphism of the *COMT* gene and the Formal Characteristics of Behavior—Temperament Inventory scores for temperamental traits were revealed. Furthermore, lower sensitivity was seen in (Val/Val) homozygous combat athletes compared to (Met/Met or Met/Val) individuals who carried the Met allele (Leźnicka et al., 2018).

## Conclusions

The present study was designed to determine the effects of the *COMT* gene rs4680 polymorphism on the psychological profile of athletes. Studying genetic associations with psychological phenotypes presents a promising avenue of inquiry regarding an athlete's success. Due to the multi-gene and multifactorial nature of the determinants of sports predispositions, we propose to take into account also other features, especially when studying genes related to cerebral neurotransmission. It is a holistic departure, and it clearly illustrates the relationship between particular characteristics of an athlete. The limitation of our research is that personality traits are not only genetically determined, but are also conditioned by the developmental environment of the athlete.

## References

[ref1] Bastos, P., Gomes, T., & Ribeiro, L. (2017). Catechol-O-Methyltransferase (COMT): An Update on Its Role in Cancer, Neurological and Cardiovascular Diseases. Reviews of Physiology, Biochemistry and Pharmacology, 173, 1–39. 10.1007/112_2017_2 28456872

[ref2] Bilder, R. M., Volavka, J., Lachman, H. M., & Grace, A. A. (2004). The catechol-O-methyltransferase polymorphism: relations to the tonic-phasic dopamine hypothesis and neuro-psychiatric phenotypes. Neuropsychopharmacology : Official Publication of the American College of Neuropsychopharmacology, 29(11), 1943–1961. 10.1038/sj.npp.1300542 15305167

[ref3] Calati, R., Porcelli, S., Giegling, I., Hartmann, A. M., Möller, H. J., de Ronchi, D., Serretti, A., & Rujescu, D. (2011). Catechol-o-methyltransferase gene modulation on suicidal behavior and personality traits: review, meta-analysis and association study. Journal of Psychiatric Research, 45(3), 309–321. 10.1016/j.jpsychires.2010.07.004 20667552

[ref4] Chen, S., Cai, W., Duan, S., Gao, L., Yang, W., Gao, Y., Jia, C., Zhang, H., & Li, L. (2021). Association of comt polymorphisms with multiple physical activity-related injuries among university students in China. International Journal of Environmental Research and Public Health, 18(20), 10828. 10.3390/ijerph182010828/S1 34682575 PMC8535648

[ref5] Cieszczyk, P., Sawczuk, M., Maciejewska, A., Ficek, K., & Eider, J. (2011). Variation in peroxisome proliferator activated receptor α gene in elite combat athletes. European Journal of Sport Science, 11(2), 119–123. 10.1080/17461391.2010.487120

[ref6] Craddock, N., Owen, M. J., & O'Donovan, M. C. (2006). The catechol-O-methyl transferase (COMT) gene as a candidate for psychiatric phenotypes: evidence and lessons. Molecular Psychiatry, 11(5), 446–458. 10.1038/sj.mp.4001808 16505837

[ref7] Guilherme, J. P. L. F., Egorova, E. S., Semenova, E. A., Kostryukova, E. S., Kulemin, N. A., Borisov, O. v., Khabibova, S. A., Larin, A. K., Ospanova, E. A., Pavlenko, A. v., Lyubaeva, E. v., Popov, D. v., Lysenko, E. A., Vepkhvadze, T. F., Lednev, E. M., Govorun, V. M., Gener-ozov, E. v., Ahmetov, I. I., & Lancha Junior, A. H. (2019). The A-allele of the FTO Gene rs9939609 Polymorphism Is Associated With Decreased Proportion of Slow Oxidative Muscle Fibers and Over-represented in Heavier Athletes. Journal of Strength and Conditioning Research, 33(3), 691–700. 10.1519/jsc.0000000000003032 30694969

[ref8] Guilherme, J. P. L. F., Souza-Junior, T. P., & Lancha Junior, A. H. (2021). Association study of performance-related polymorphisms in Brazilian combat-sport athletes highlights variants in the GABPB1 gene. Physiological Genomics, 53(2), 47–50. 10.1152/physiolgenomics.00118.2020 33346691

[ref9] Haraldsson, H. M., Ettinger, U., Magnusdottir, B. B., Sigmundsson, T., Sigurdsson, E., Ingason, A., & Petursson, H. (2010). Catechol-O-Methyltransferase Val158Met Polymorphism and An-tisaccade Eye Movements in Schizophrenia. Schizophrenia Bulletin, 36(1), 157. 10.1093/schbul/SBN064 18562342 PMC2800134

[ref10] Harrison, P. J., & Tunbridge, E. M. (2008). Catechol-O-methyltransferase (COMT): a gene contributing to sex differences in brain function, and to sexual dimorphism in the predisposition to psychiatric disorders. Neuropsychopharmacology : Official Publication of the American College of Neuropsychopharmacology, 33(13), 3037–3045. 10.1038/sj.npp.1301543 17805313

[ref11] Humińska-Lisowska K, Chmielowiec J, Chmielowiec K, Niewczas M, Lachowicz M, Cięszczyk P, Masiak J, Strońska-Pluta A, Michałowska-Sawczyn M, Maculewicz E, Grzywacz A. (2022) Associations of Brain-Derived Neurotropic Factor rs6265 Gene Polymorphism with Personality Dimensions among Athletes. International Journal of Environmental Research and Public Health, 19(15), 9732. 10.3390/ijerph19159732 35955088 PMC9367731

[ref12] Jaspar, M., Genon, S., Muto, V., Meyer, C., Manard, M., Dideberg, V., Bours, V., Salmon, E., Maquet, P., & Collette, F. (2014). Modulating effect of COMT genotype on the brain regions underlying proactive control process during inhibition. Cortex, 50, 148–161. 10.1016/j.cortex.2013.06.003 23859480

[ref13] Kochanowicz, A., Kochanowicz, K., Niespodziński, B., Mieszkowski, J., & Biskup, L. (2015). The level of body balance in a handstand and the effectiveness of sports training in gymnastics. Baltic Journal of Health and Physical Activity, 7(4), 117–124. doi: 10.29359/bjhpa.07.4.11

[ref14] Leźnicka, K., Kurzawski, M., Ciȩszczyk, P., Safranow, K., Malinowski, D., Brzeziańska-Lasota, E., & Zmijewski, P. (2017). Polymorphisms of catechol-O-methyltransferase (COMT rs4680:G>A) and μ-opioid receptor (OPRM1 rs1799971:A >G) in relation to pain perception in combat athletes. Biology of Sport, 34(3), 295–301. 10.5114/biolsport.2017.67856

[ref15] Leźnicka, K., Niewczas, M., Kurzawski, M., Cięszczyk, P., Safranow, K., Ligocka, M., & Białecka, M. (2018). The association between COMT rs4680 and OPRM1 rs1799971 polymorphisms and temperamental traits in combat athletes. Personality and Individual Differences, 124, 105–110. 10.1016/j.paid.2017.12.008

[ref16] Machoy-Mokrzyńska, A., Starzyńska-Sadura, Z., Dziedziejko, V., Safranow, K., Kurzawski, M., Leźnicka, K., Sulżyc-Bielicka, V., Jurewicz, A., Bohatyrewicz, A., & Białecka, M. (2019). As-sociation of COMT gene variability with pain intensity in patients after total hip replacement. Scandinavian Journal of Clinical and Laboratory Investigation, 79(3), 202–207. 10.1080/00365513.2019.1576920 30822160

[ref17] Maciejewska-Skrendo, A., Sawczuk, M., Cięszczyk, P., & Ahmetov, I. (2019). Chapter Three – Genes and power athlete status. In Barh D & Ahmetov II (Eds.), Sports, Exercise, and Nutritional Genomics. (pp. 41–72). Academic Press.

[ref18] Nogueira, N. G. de H. M., Bacelar, M. F. B., Ferreira, B. de P., Parma, J. O., & Lage, G. M. (2019). Association between the catechol-O-methyltransferase (COMT) Val 158 Met polymorphism and motor behavior in healthy adults: A study review. Brain Research Bulletin, 144, 223–232. 10.1016/j.brainresbull.2018.11.002 30445182

[ref19] Niewczas, M., Grzywacz, A., Leźnicka, K., Chmielowiec, K., Chmielowiec, J., Maciejewska-Skrendo, A., Ruzbarsky, P., Masiak, J., Czarny, W., & Cięszczyk, P. (2021). Association between Polymorphism rs1799732 of DRD2 Dopamine Receptor Gene and Personality Traits among MMA Athletes. Genes, 12, 1217. 10.3390/genes12081217 34440391 PMC8391442

[ref20] Perkovic, M. N., Strac, D. S., Tudor, L., Konjevod, M., Erjavec, G. N., & Pivac, N. (2018). Cate-chol-O-methyltransferase, Cognition and Alzheimer's Disease. Current Alzheimer Research, 15(5), 408–419. 10.2174/1567205015666171212094229 29231139

[ref21] Ribas, M. R., Netto, Z. C. O., Salgueirosa, F., Fernandes, P., de Matos, O., & Bassan, J. C. (2017). Association of ACTN3 r577x and ACE i/d polymorphisms in Brazilian wrestlers. Revista Brasileira de Medicina Do Esporte, 23(6), 469–472. 10.1590/1517-869220172306171864

[ref22] Savitz, J., Solms, M., & Ramesar, R. (2006). The molecular genetics of cognition: dopamine, COMT and BDNF. Genes, Brain, and Behavior, 5(4), 311–328. 10.1111/J.1601-183X.2005.00163.X 16716201

[ref23] Srivastava, K., Ochuba, O., Sandhu, J.K., Alkayyali, T., Ruo, S.W., Waqar, A., Jain, A., Joseph, C., & Poudel, S. (2021). Effect of Catechol-O-Methyltransferase Genotype Polymorphism on Neurological and Psychiatric Disorders: Progressing Towards Personalized Medicine. Cureus, 13(9), e18311. 10.7759/cureus.18311 34725583 PMC8553290

[ref24] Switala, K., & Leonska-Duniec, A. (2021) Physical activity and gene association with human obesity. Baltic Journal of Health and Physical Activity, 13(4), 99–111. doi: 10.29359/bjhpa.13.4.10

[ref25] Tartar, J. L., Cabrera, D., Knafo, S., Thomas, J. D., Antonio, J., Peacock, C. A. (2020). The “Warrior” COMT Val/Met genotype occurs in greater fre-quencies in mixed martial arts fighters relative to controls. Journal of Sports Science and Medicine, 19(1), 38–42. 32132825 PMC7039020

[ref26] van Breda, K., Collins, M., Stein, D. J., & Rauch, L. (2015). The COMT val(158)met polymorphism in ultra-endurance athletes. Physiology & Behavior, 151, 279–283. 10.1016/j.physbeh.2015.07.039 26253211

[ref27] Weinshilboum, R. M., Otterness, D. M., & Szumlanski, C. L. (1999). Methylation pharmacogenetics: catechol O-methyltransferase, thiopurine methyltransferase, and histamine N-methyltransferase. Annual Review of Pharmacology and Toxicology, 39, 19–52. 10.1146/annurev.pharmtox.39.1.19 10331075

[ref28] Youn, B. Y., Ko, S. G., & Kim, J. Y. (2021). Genetic basis of elite combat sports athletes: a systematic review. Biology of Sport, 38(4), 667–675. 10.5114/biolsport.2022.102864 34937977 PMC8670794

[ref29] Zmijewski, P., Leońska-Duniec, A., Stuła, A., & Sawczuk, M. (2021). Evaluation of the Association of COMT Rs4680 Polymorphism with Swimmers' Competitive Performance. Genes, 12(10), 1641. 10.3390/genes12101641 34681035 PMC8535192

